# 

**DOI:** 10.1017/psy.2025.10051

**Published:** 2025-11-25

**Authors:** Edanur Dayıoğlu

**Affiliations:** Bursa Uludağ University, Bursa, Turkiye

Complex systems that accompany us at every moment of our daily lives reveal the fundamental dynamics that shape our lives, even if we do not consciously perceive them (Hmelo-Silver & Pfeffer, 2004). Although there is no generally accepted formal definition of complex systems, which have been studied extensively from the 19th century to the present, Mitchell (2006) defined complex systems as a large network consisting of simple elements to some extent, without a central control, and stated that complex behaviors emerge as a result of the interaction of the elements in this network. Another definition is provided by Maguire and McKelvey (1999) defined complex systems as systems composed of various components, each acting according to rules, forces, and laws within its own local context. Many mathematical and computational models have been developed to study complex systems, and the comprehensive, quantitative, and reliable data from natural sciences have been used to test these models. However, since not all data in the behavioral and social sciences can be easily quantified, applying complex system methodologies to these fields is considered more challenging compared to natural sciences. Therefore, complex systems have made slower progress in the field of behavioral and social sciences compared to natural sciences (van der Maas, 2024).

Although the application of complex systems to behavioral sciences is challenging, van der Maas (2024) argued that the human brain is itself a large complex system. Therefore, humans can be considered the largest complex system, which makes it imperative to apply the complex systems approach to behavioral sciences. Within the field of psychology, the complex systems approach focuses on explaining the dynamics resulting from the interactions of individuals or groups in the context of mental and behavioral processes (Holland, 2014; Tononi, 2004). This review explores how complex systems are addressed in psychology, examining van der Maas’s work in terms of both content and methodology. Its contributions and limitations to the field of psychology are analyzed within an analytical framework. In particular, this review examines the book’s compatibility with its target audience, its treatment of theoretical approaches, and its practical value for professional application are examined. In his context, the review begins with general information about the author and the book. Next, it examines the chapters by comparing them with relevant studies in the literature. Finally, the review will discuss the book’s strengths and weaknesses.

Han L. J. van der Maas, a Professor of Psychological Methods at the University of Amsterdam’s Department of Psychology, conducts research on complex systems in the behavioral and social sciences. The book titled *Complex Systems Research in Psychology*, written by van der Maas, was published as an open access on August 30, 2024 by SFI Press, a non-profit organization affiliated with the Santa Fe Institute, which was established to conduct research in the field of complex systems. This book, which explores how complex systems are applied in psychology, has three main objectives: to provide a comprehensive perspective on complex systems, to develop research skills for complex systems research, and to encourage critical thinking about the potential applications of complex systems in psychology. Van der Maas stated that his target while writing the book included psychologists and social scientists who are interested in modeling psychological processes using complex systems research tools. However, van der Maas also stated that the information in the book is mostly for psychologists who have limited knowledge about complex systems, although graduate students in psychology who are conducting research in psychometrics, cognitive psychology, social psychology, and methodology may likewise find it beneficial. As the book is written with a high level of theoretical depth and adopts an interdisciplinary perspective, readers are expected to have a certain level of knowledge in the fields of quantitative research methodology, including statistical modeling and mathematical backgrounds, psychology, R language, and mathematics.

van der Maas structured the book titled *Complex Systems Research in Psychology* into seven chapters, each designed to complement the others by presenting essential background information. The content was evaluated based on teaching principles; the principle of easy to difficult in terms of difficulty, the principle of known to unknown, where information related to the subject is reviewed before moving on to new information (Gökalp, 2019). The first chapter of the book covers the general definition and basic concepts of complex systems. By giving the example of the synchronized movements of bird flocks, the characteristics of complex systems, such as self-organization, unpredictable behavior, and patterns that emerge as a result of the interactions of subsystems, are explained. This aligns with the principle of “disorder creates order” advocated by Prigogine (1997). Additionally, the first chapter of the book addresses the concept of emergentism, explaining the nonlinear nature of psychological processes, and focuses on applications in the field of psychology and methods for analyzing complex systems. It also explores the modeling of complex systems as networks, a topic often framed in the context of Barabási’s (2002) work on network theory, and considers how psychological systems exhibit organization within disorder. In the second chapter of the book, the basic principles of chaos theory and chaotic elements in psychological processes are discussed, along with detailed explanations of concepts, such as modeling population growth with logistic maps, stable and unstable fixed points, limit cycles, and phase plots. The applicability of chaos theory in the field of psychology is examined, and examples illustrate how chaos can be detected in psychophysiological data. Guastello’s (1995) insights regarding “Chaotic Dynamics and Psychology” and his approach to the applicability of chaos theory to psychology are closely aligned with the arguments presented in the book.

In the third chapter, where phase transitions, bifurcation theory, and catastrophe theory are discussed in more detail, the mathematical models of abrupt changes in complex systems and their effects on psychological processes are examined. Additionally, the observability of these transitions is evaluated through examples such as the Necker cube. The ideas presented in this section also overlap with the Dynamic Pattern Theory developed by Kelso (1995). Kelso’s studies provide important contributions to understanding dynamic transitions in psychological processes by examining synchronization changes in neurological processes. However, despite the availability of relevant experimental studies by Kelso, no modeling is presented in this section within the context of a psychological experiment.

The fourth chapter discusses how dynamic system models can be used in the temporal analysis of psychological processes. The fundamental properties of dynamic systems are introduced. The importance of using dynamic system models in social sciences is emphasized, and it details tools, such as psychological models and causal-loop diagrams. This section parallels the “Dynamic Systems Theory” developed by Thelen and Smith (1994) and offers an additional perspective on the approaches used in modeling psychological processes and how dynamic system models can be used in motor development and cognitive processes.

Self-organization, one of the fundamental properties of complex systems, is explained in the fifth chapter with examples from different disciplines, such as physics, chemistry, biology, and neural networks. This chapter provides concise, step-by-step guidance for various modeling procedures, including examples from multiple disciplines. Statistical inferences are explicitly addressed under each topic, emphasizing their relevance for understanding both individual and social systems. It addresses the role of self-organization in psychological and social systems using mathematical models, such as the NetLogo programming language, the Ising Model, and the Game of Life. The concept of self-organization is also linked to Haken’s (1983) theory of “Synergetic Dynamics.” This theory explains the synchronization of the system without external intervention and is applied to understanding organizational changes in individual or group dynamics. The chapter underlines the interdisciplinary nature of self-organization—originally rooted in the natural sciences but now applied to psychology, where quantitative methods like network approaches and agent-based simulations capture emergent dynamics. This interdisciplinary theme allows quantitative psychologists to formalize theories, advance network modeling, and apply new methods to issues, such as psychopathology and collective decision-making. By connecting synergetic dynamics to psychological modeling, the chapter shows that self-organization offers a unifying framework and methodological bridge, enabling psychologists to enrich theory and practice by drawing on tools from the natural sciences.

The sixth chapter addresses the role of network models in understanding psychological phenomena. It discusses how psychological network models can represent the mental and behavioral interactions of individuals as an alternative to traditional cause–effect models. It discusses the techniques that can be used in psychometric data analysis, as well as the theoretical and methodological challenges faced by these models. The “Psychological Network Models” developed by Borsboom and Cramer (2013) provide a theoretical basis for the book’s approach by offering a different approach than traditional factor analysis. This chapter illustrates, through examples, how network-based approaches, such as the mutualism model and the Ising attitude model, explain complex psychological processes, including the dynamic development of intelligence and attitudes, as well as the mutual interactions of symptoms. It also explains the use of contemporary psychometric network techniques—such as Gaussian graphical models, partial correlation networks, and mixed graphical models—highlighting these methods as a strong alternative to traditional factor analysis. However, the book only provides a general overview.

The seventh and final chapter of the book focuses on understanding the dynamics between individuals and explains the concept of sociophysics. It emphasized the similarity of the characteristics of complex systems, such as phase transition and unpredictability in social systems to social dynamics. The chapter also explores how individuals’ views and behaviors evolve over time drawing on examples, such as the Schelling Model and the Similarity Tendency (homophily). The arguments in the book support the idea that social behaviors can be modeled in a similar way to physical systems, paralleling Galam’s (2008) understanding of sociophysics. Throughout these chapters, the book makes several contributions to the literature by examining how complex systems theory is applied to psychology, while also leaving certain aspects of the subject open for further exploration.

At the end of each chapter, the inclusion of exercises as the last heading (modeling with tools, such as R and NetLogo) and the presentation of seminars, applications, books, and websites as examples for readers to gain more information on the topics increase the comprehensibility of the related concepts. The book discusses the applications of complex systems to the field of psychology from a broad perspective with phenomena, such as network theory and dynamical systems and reinforces the topics with examples from different disciplines, such as physics, biology, and computer science. It also touches on how different disciplines can benefit from each other in complex systems studies providing relevant examples. It contributes to the fields of cognitive psychology and neuroscience by modeling cognitive processes in the context of complex systems. It also aims to bridge methodologically between different disciplines of psychology by addressing the way individual-based cognitive processes combine with collective behavior.

On the other hand, the book’s heavy reliance on abstract mathematical language, with minimal use of psychological terminology or illustrative examples, significantly hinders its accessibility. This lack of contextualization renders the content especially difficult for social science readers, limiting its usefulness beyond a quantitatively specialized audience. Furthermore, the validation of the models presented in the book with psychological data is not mentioned much, and how models can be reconciled with psychological experiments is not sufficiently discussed. The book does not discuss the relationship between sub-fields of psychology (e.g., clinical or developmental psychology) and complex systems, nor does it address specific applications to these fields. It also lacks case studies illustrating how the presented models apply to real-world problems encountered in daily life.

In general, this book presents an interdisciplinary approach to psychology by applying a complex systems approach to psychology and provides an overview of different methods that can be used in psychology. It particularly attempts to offer a new perspective to understand interpersonal dynamics and processes by going beyond traditional variable-centered approaches and traditional linear models in psychology. However, this framework needs more concrete examples and verification with psychological data to make this framework practically applicable. Integrating findings from recent empirical studies in the field of psychology could further enhance its relevance and utility.
